# Disabling osteomalacia and myopathy as the only presenting features of celiac disease: a case report

**DOI:** 10.1186/1757-1626-2-20

**Published:** 2009-01-07

**Authors:** Costantine Albany, Zhanna Servetnyk

**Affiliations:** 1 Department of Medicine, St. Luke's Roosevelt Hospital Center, Columbia University College of Physicians and Surgeons, 1000 Tenth Avenue New York, NY 10019 USA

## Abstract

**Background:**

Celiac disease is an autoimmune inflammatory disease of the small intestine precipitated by the ingestion of gluten, a component of wheat protein, in genetically susceptible persons. Classically, the disease manifests with diarrhea, weight loss and anemia. There are very few reports of osteomalacia as the presenting symptom, and even fewer of osteomalacia as the only symptom of celiac disease at presentation.

**Case presentation:**

In this case report we describe a 40 year-old patient who presented with 6 months history of progressively worsening and disabling osteomalacia as the only presentation of a celiac disease. With adherence to a gluten-free diet and calcium-vitamin D supplementation, the patient's condition improved remarkably within three months and she was able to walk pain free.

**Conclusion:**

Celiac disease frequently is under diagnosed or misdiagnosed which results in unnecessary morbidity such as disabling osteomalacia. Therefore, early diagnosis of celiac disease is important because the symptoms completely resolve with adequate treatment.

## Background

Celiac disease (CD) is a chronic digestive disease in which patients have inflammation in the small intestine. Inflammation in the bowel occurs when a patient with CD begins to eat food that contains gluten. Approximately 1 out of every 250 people may have CD though only 1 out of 10 people with celiac disease actually may be diagnosed and are aware of their disease. Some of these patients have mild forms of CD and may be asymptomatic or have only mild symptoms. Classically, the disease manifests with diarrhea, weight loss and anemia. There are very few reports of osteomalacia as the presenting symptom, and even fewer of osteomalacia as the only symptom of celiac disease at presentation [1,2].

## Case presentation

A 40-year-old Caucasian woman, with past medical history significant for depression, presented with a chief complain of diffuse bone pain and proximal muscular weakness, mainly in the lower extremities and to a less extent in both shoulders for six months. The pain and weakness progressed to the point that she had difficulties in rising from a chair, holding her arms up and walking, and inability to ascend stairs.

Clinical examination showed evidence of bilateral, proximal muscle atrophy and weakness in the upper and lower extremities, and hypoactive reflexes in four extremities. Her hip range of motion was limited and painful. She had a waddling gait pattern. Laboratory workup (Table 1) revealed microcytic anemia, hypocalcemia, hypophosphoremia and raised serum alkaline phosphatase. Vitamin D (25-OH vitD) was very low. Other laboratory investigations were normal, including erythrocyte sedimentation rate (ESR), C-reactive protein (CRP), thyroid function tests, and creatinine kinase. Bone mineral density using dual energy X-ray absorptiometry (DEXA) scan was done and T score measurements were low (3.5 SD below the mean at the femoral neck and 2.5 SD below the mean at the lumbar spine). The diagnosis of osteomalacia was made. Investigations for malabsorption were carried out and IgA antiendomysial antibody and antigliadin IgA and IgG antibodies were found to be elevated.

**Table 1:** Laboratory workup

Chemistry			Hematology		
Calcium	7.6 mg/dl	(8–10.6 mg/dl)	WBC	6.4 K/uL	(4.5–10.8 K/uL)
Phosphorus	1.9 mg/dl	(2.5–4.5 mg/dl)	Hemoglobin	9.96 g/dl	(12–16 g/dl)
Alkaline Phosp	866 U/l	(50–305 U/l)	Hematocrit	30%	(40–51%)
25-OH vitamin D	10 ng/ml	(14–75 ng/ml)	Platelet	322 K/uL	(150–450 K/uL)

Esophagogastroduodenoscopy (EGD) with distal duodenal biopsy confirmed a diagnosis of celiac disease. With adherence to a gluten-free diet and calcium-vitamin D supplementation, the patient's condition improved remarkably within three months and she was able to walk pain free.

## Discussion

Celiac disease is a disorder with a genetic predisposition that results in hypersensitivity to the gliadin fraction of gluten [3]. The disease is common among Caucasians. Recent studies in the United States suggest that the prevalence of celiac disease is approximately 1 case per 250 persons [4]. There may be as many as 1 million people in the United States and 3–5 million in the world with CD [5]. Women comprise approximately 75 percent of newly diagnosed adult celiac disease cases. Women also tend to have more clinically prominent disease [6]. In adults, gastrointestinal tract involvement may manifest as diarrhea, constipation, or other symptoms of malabsorption, such as bloating, flatus, or belching. Rarely, celiac disease presents with different systemic manifestations, which include osteomalacia, osteoporosis, anemia of various types, dermatitis herpetiformis, depression, dementia, and dental enamel defects [7,8].

This patient presented with moderate osteomalacia in the absence of any gastrointestinal symptoms. An association between celiac disease and osteomalacia was first reported in 1953[9]. The clinical presentation with lone symptomatic osteomalacia is very unusual in celiac disease and few cases are described in the literature. [1,10]

The diagnosis of celiac disease is based on clinical, serological and endoscopic evaluation [11]. IgA antiendomysial antibody has been shown to be 85 to 100 percent sensitive and 96 to 100 percent specific for celiac disease. Hypocalcaemia in celiac disease – as in this case – is related to reduced gut absorption of calcium due to reduced levels of vitamin D; it is also due to reduced absorptive surface area secondary to villous atrophy [12]. Distal duodenal biopsy is the gold standard for the diagnosis of celiac disease. Biopsy should be performed in most patients with suspected gluten-sensitive enteropathy [5].

Since celiac disease is the second most common cause of osteomalacia after gastrectomy in the United States, it is very important to consider the diagnosis in patients presenting with either osteomalacia or osteoporosis. Also bone density should be assessed in all patients with newly diagnosed celiac disease to evaluate for osteoporosis and osteomalacia.

The primary treatment for celiac disease is the removal of gluten and related proteins from the diet. Exclusion of dietary gluten generally results in rapid and complete healing of small-bowel inflammation. Advice from a registered dietitian is essential to outline an appropriate diet. Osteomalacia in adults starts insidiously as aches and pains in the lumbar region and thighs, spreading later to the arms and ribs. Pain is non-radiating, symmetrical, and accompanied by tenderness in the involved bones. Proximal muscles are weak, and there is difficulty in climbing up stairs and getting up from a squatting position [13]. Osteomalacia results from vitamin D deficiency. It should be treated with calcium and vitamin D replacement; luckily this condition is reversible and treatment leads to remineralization of the skeleton. Celiac disease frequently is under diagnosed or misdiagnosed which results in unnecessary morbidity such as disabling osteomalacia and fractures. Therefore, early diagnosis of celiac disease is important because the symptoms completely resolve with adequate treatment.

## Abbreviations

CD: Celiac Disease; ESR: Erythrocyte Sedimentation Rate; CRP: C-reactive protein; DEXA: Dual energy X-ray absorptiometry.

## Consent

Written informed consent was obtained from the patient for publication of this case report. A copy of this written consent is available for review by the Editor-in-chief of this journal.

## Competing interests

The authors declare that they have no competing interests.

## Authors' contributions

CA and ZS contributed equally in writing the manuscript. Both authors read and approved the final manuscript.
